# Nucleotide excision repair is a predictor of early relapse in pediatric acute lymphoblastic leukemia

**DOI:** 10.1186/s12920-018-0422-2

**Published:** 2018-10-30

**Authors:** Omar M. Ibrahim, Homood M. As Sobeai, Stephen G. Grant, Jean J. Latimer

**Affiliations:** 1 Department of Pharmaceutical Sciences, College of Pharmacy, 3200 S University Drive, Fort Lauderdale, FL 33328 USA; 2 AutoNation Institute for Breast and Solid Tumor Cancer Research, 3301 College Avenue, Fort Lauderdale, FL 33314 USA; 3 Department of Public Health, Dr. Kiran C. Patel College of Osteopathic Medicine, Nova Southeastern University, 3200 S University Drive, Fort Lauderdale, FL 33328 USA; 4 Department of Pharmacology and Toxicology, College of Pharmacy, King Saud University, P.O. Box 2475, Riyadh, 11451 Saudi Arabia

**Keywords:** Nucleotide excision repair gene, Acute lymphoblastic leukemia, Childhood ALL, Precursor-B-ALL, Early relapse, Late relapse

## Abstract

**Background:**

Nucleotide Excision Repair (NER) is a major pathway of mammalian DNA repair that is associated with drug resistance and has not been well characterized in acute lymphoblastic leukemia (ALL). The objective of this study was to explore the role of NER in relapsed ALL patients. We hypothesized that increased expression of NER genes was associated with drug resistance and relapse in ALL.

**Methods:**

We performed secondary data analysis on two sets of pediatric ALL patients that all ultimately relapsed, and who had matched diagnosis-relapse gene expression microarray data (GSE28460 and GSE18497). GSE28460 included 49 precursor-B-ALL patients, and GSE18497 included 27 precursor-B-ALL and 14 T-ALL patients. Microarray data were processed using the Plier 16 algorithm and the 20 canonical NER genes were extracted. Comparisons were made between time of diagnosis and relapse, and between early and late relapsing subgroups. The Chi-square test was used to evaluate whether NER gene expression was altered at the level of the entire pathway and individual gene expression was compared using *t*-tests.

**Results:**

We found that gene expression of the NER pathway was significantly increased upon relapse in patients that took 3 years or greater to relapse (late relapsers, *P = .*007), whereas no such change was evident in patients that relapsed in less than 3 years (early relapsers, *P =* .180). Moreover, at diagnosis, the NER gene expression of the early relapsing subpopulation was already significantly elevated over that of the late relapsing group (*P < .001*). This pattern was validated by an ‘NER score’ established by averaging the relative expression of the 20 canonical NER genes. The NER score at diagnosis was found to be significantly associated with disease-free survival in precursor-B-ALL (*P* < .001).

**Conclusion:**

Patients are over two times more likely to undergo early relapse if they have a high NER score at diagnosis, hazard ratio 2.008, 95% CI (1.256–3.211). The NER score may provide a underlying mechanism for “time to remission”, a known prognostic factor in ALL, and a rationale for differential treatment.

**Electronic supplementary material:**

The online version of this article (10.1186/s12920-018-0422-2) contains supplementary material, which is available to authorized users.

## Introduction

Acute lymphoblastic leukemia (ALL) is the most common childhood cancer, however, treatment has improved dramatically due to the ability to stratify patients into groups based on risk factors and genetic analysis [1–3]. By designing group-specific treatment protocols, pediatric ALL 5-year relative survival rates have increased substantially from 57% in 1975–1977 to 92% in 2006–2012 [4–7]. Newly diagnosed ALL patient treatment relies heavily on traditional chemotherapeutic agents, such as vincristine, anthracyclines, methotrexate, mercaptopurine, thioguanine, cyclophosphamide, and cytarabine [8].

The basis of genotoxic chemotherapy is to induce DNA damage in rapidly growing cells to critically damage and eradicate the cancer [9]. However, cancers can clonally or epigenetically acquire elevated DNA repair capacity and more efficiently remediate chemotherapy-induced genotoxic damage, thus allowing the cancer to continue growing [10, 11]. The study of DNA repair in ALL has been limited to homologous recombination and non-homologous end joining, with little investigation of base repair mechanisms such as nucleotide excision repair (NER) [12–19]. NER is a versatile DNA repair pathway and is involved in remediating damage induced by anthracyclines, cyclophosphamide and other agents [20, 21]. This pathway requires 20 canonical proteins to complete repair [22]. There is little to no information on the role of NER in childhood ALL resistance or relapse.

15–20% of ALL patients who have reached complete remission eventually relapse. ALL relapse is considered one of the major cancer-related causes of death among childhood malignancies [3, 23–25]. Relapse can occur even in patients with favorable prognostic factors at diagnosis [26]. Time to relapse is an important factor in predicting the successful treatment of relapsed ALL. Thus, identifying patients with highest risk of early recurrence who are not detectable using current clinical measures and treating them with a more targeted therapy is crucial to overcoming this problem. Cytogenetics are often used to help determine the prognosis of a precursor-B-ALL diagnosis; however, the role of NER is unclear. The aim of the current study is to investigate the gene expression of NER in relapsing childhood ALL by secondary analysis of two published data sets and to determine its potential as a prognostic factor.

## Methods

### Patient samples

This study included two matched diagnosis-relapse paired gene expression datasets of pediatric ALL (GSE18497, Staal et al. and GSE28460, Hogan et al.) [27, 28] available on the Gene Expression Omibus website (GEO) by NCBI. Patient information from both databases were summarized in Additional file 1: Table S1. The Staal dataset included 41 patients diagnosed with both precursor-B-ALL (*n* = 27) and T-ALL (*n* = 14) treated with Dutch Childhood Oncology Group protocols (1989–2004) [29]. The Hogan dataset included 49 patients, precursor-B-ALL treated using Children’s Oncology Group (COG) protocols [30]. In the Staal data set only information from precursor-B-ALL patients were analyzed to remain consistent with the Hogan dataset. Both sets of protocols included chemotherapeutic regimens that could select for drug resistant cells due to changes in NER gene expression.

### Microarray and data processing

The Affymetrix Human Genome U133 plus 2 array was used to generate both datasets. Gene expression (.CEL) files were downloaded from GEO, and then processed using Genespring software (Affymetrix). The probe logarithmic intensity error (PLIER16) algorithm was applied to normalize dataset files [31]. Data on 51 probes representing 20 NER canonical genes were extracted. Expression data on multiple probes for a single gene were averaged.

### Patient stratification

The expression of the canonical 20 NER genes was examined in matched pediatric samples at the time of diagnosis and relapse of only precursor-B-ALL patients from both datasets. [32] We classified patients based on the time of recurrence as either early (< 36 months) or late (≥36 month) relapsers, regardless of other prognostic variables. Four NER subgroup analyses were conducted: matched diagnosis-relapse samples of early relapsers; matched diagnosis-relapse samples of late relapsers; early vs. late relapsers at the time of diagnosis; early vs. late relapsers at the time of relapse.

At the end of each analysis, Hogan and Staal datasets were combined after normalizing the data to a reference (control) group in each dataset. The reference (control) groups were NER gene expression values at the time of diagnosis in diagnosis-relapse matched analyses or the expression data in late relapsing patients at the time of diagnosis and at the time of recurrence.

### Statistical analysis

The Chi-square test was used to evaluate whether NER gene expression was altered at the level of the whole pathway. The proportion of upregulated NER genes was compared to a theoretical “no change” prediction of 10 genes upregulated and 10 genes downregulated. When comparing between actual datasets, Fisher’s exact test was used. We also performed a one-tailed paired (diagnosis vs. relapse) or unpaired Student’s *t* test (subset analyses of early and late relapsers) to identify individual NER genes that were significantly increased in expression between groups.

NER scores were generated by averaging the expression of all NER genes relative to that of the average of all patients at relapse for each respective dataset. Paired and unpaired *t* tests were used to evaluate differences in NER scores at diagnosis vs. relapse and between early and later relapsers, respectively. The Chi-square test was used to determine the significance of skewing of the distribution of paired patient diagnosis-relapse NER scores.

Disease-free survival curves of NER score at time of diagnosis were plotted using Kaplan-Meier (KM) plots for each dataset to assess the ability of the NER pathway to be used prognostically. NER scores in KM plots used information from diagnosis only and were expressed relative to the average of relapse for each dataset. The patients were then divided into two groups based on their score: patients with an NER score above the average at diagnosis were placed in the high group and patients below the average were placed in the low group. KM curves were created using the statistical software Prism 7.0 (GraphPad Software, Inc.). The log-rank test and log-rank hazard ratio (HR) tests were used to measure significance between the two KM curves for each plot. A *P* value of < .05 was considered statistically significant. NER score with other cytogenetic variables were compared in Additional file 2: Figure S1 and Additional file 3: Figure S2.

## Results

### Diagnosis vs. relapse paired samples

**Fig. 1:**
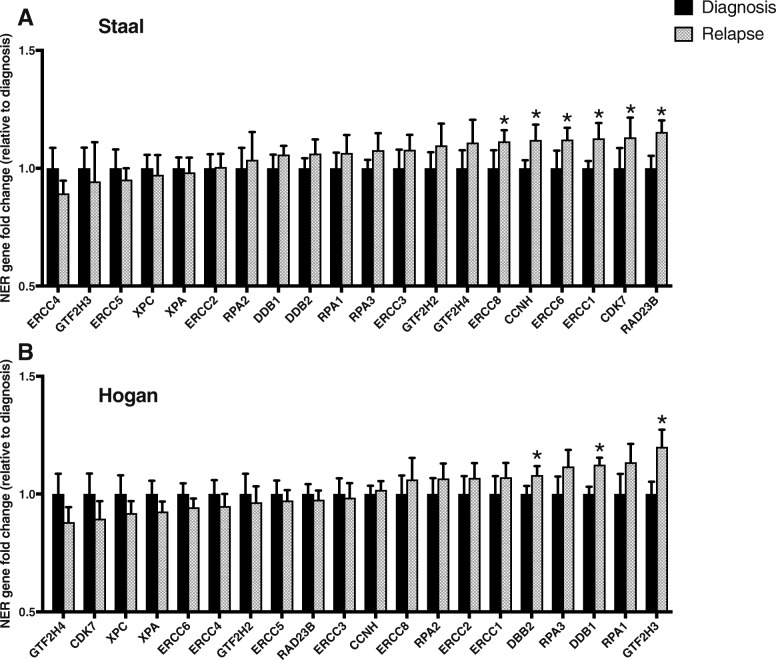
Comparative expression of the 20 canonical NER genes at diagnosis versus relapse. (A) In the Staal dataset (*n* = 27), 15 genes had higher expression at the time of relapse (gray bars) than at the time of diagnosis (black bars) (*P* = .006). 6 of these genes were individually significant: *ERCC8* (*P* = .010), *CCNH* (*P* = .047), *ERCC6* (*P* = .013), *ERCC1* (*P* = .018), *CDK7* (*P* = .020), and *RAD23B* (*P* = .022). (B) In the Hogan dataset (*n* = 49), 10 genes were overexpressed at time of relapse compared to at time of diagnosis (*P* = 1.000). 3 genes were individually significantly upregulated: *DDB2* (*P* = .044), *DDB1* (*P* < .001), and *GTF2H3* (*P* = .005). * indicates *P* < .05 in a one tailed *t*-test

We compared NER gene expression in the Staal dataset at the time of diagnosis to time of relapse. In this dataset, we compared paired samples from 27 patients with precursor-B-ALL; we found that 15/20 NER genes had higher expression at the time of relapse than at the time of diagnosis (Fig. 1a). The overall NER pathway was therefore significantly upregulated, based on the Chi-square test (*P* = .025). The increase in expression of six of these genes was individually significant. In the Hogan dataset (49 patients, precursor-B-ALL), we found that 10/20 NER genes were overexpressed at time of relapse compared to time of diagnosis (Fig. 1b), indicating that there was no significant change in the overall expression of the pathway (*P* = 1.0), although three genes were individually significantly upregulated. The increase observed in the Staal data set was therefore not validated in the Hogan dataset for changes in NER from diagnosis to relapse.

### Early and late relapse subgroups across time (at diagnosis vs. relapse)

**Fig. 2:**
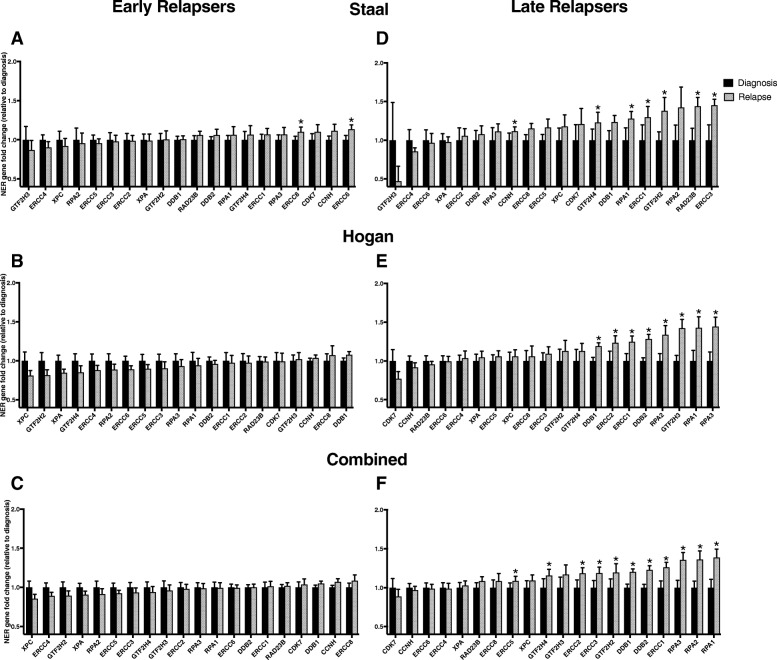
Subgroups of Early and Late Relapsers compared at the time of diagnosis to relapse. (A) In early relapsers of the Staal dataset (*n* = 19) 12 genes were overexpressed at time of relapse (gray bars, *P* = .371) versus time of diagnosis (black bars). 2 genes were individually significant *ERCC8* (*P* = .042) and *ERCC6* (*P* = .019). (B) In early relapsers of the Hogan dataset (*n* = 27), 4 genes had an increased in gene expression. The pathway was significantly under-expressed (*P* = .007). No genes were significantly overexpressed. (C) In the combined early relapsers dataset (*n* = 46), 7 genes were upregulated at the time of recurrence (*P* = .180). (D) In the late relapsers of the Staal dataset (*n* = 8) 16 NER genes were upregulated at the time of relapse versus diagnosis (*P* = 0.007). 7 genes were individually significantly overexpressed, *CCNH* (*P* = .044), *GTF2H4* (*P* = .049), *RPA1* (*P* = .030), *ERCC1* (*P* = .035), *GTF2H2* (*P* = .033), *RAD23B* (*P* = .040), *ERCC3* (*P* = .034). In the late relapsers of the Hogan dataset (*n* = 22), 16 genes were overexpressed (*p* = .007). 8 genes were individually significantly overexpressed, *DDB1* (*P* = .003), *ERCC2* (*P* = .021), *ERCC1* (*P* = .015), *DDB2* (*P* < .001), *RPA2* (*P* = .005), *RPA1* (*P* = .004), *GTF2H3* (*P* < .001), and *RPA3* (*P* < .001). (F) In the late relapsers of the combined dataset (*n* = 30), 16 genes were overexpressed at recurrence (*P* = .007) versus diagnosis. 11 genes showed significantly increased expression at relapse: *ERCC5* (*P* = .035), *GTF2H4* (*P* = .025), *ERCC2* (*P* = .013), *ERCC3* (*P* = .016), *GTF2H2* (*P* = .012), *DDB1* (*P* < .001), *DDB2* (*P* < .001), *ERCC1* (*P* < .001), *RPA3* (*P* < .001), *RPA2* (*P* < .001), and *RPA1* (*P* < .001). * indicates *P* < .05 in a one tailed *t*-test

Time to relapse is an important factor in predicting the successful treatment of relapsed ALL. We have rationalized that based on timing of relapse different subtypes of leukemia may account for differences in NER gene expression not seen in our previous analysis. We therefore investigated NER gene expression for its role in the timing of ALL relapse by dividing the data into groups based on when they initially relapsed. After patient stratification into early (< 36 months) and late (≥36 month) relapsers, NER gene expression at the time of diagnosis was compared to expression at the time of recurrence in each subgroup. In the Staal dataset, we found in the early relapsers 12/20 NER genes were upregulated at relapse when compared to diagnosis (Fig. 2a), including two genes that were individually significantly upregulated. There was no trend towards overexpression of the entire NER pathway (*P* = .371). In the early relapsers of the Hogan dataset (Fig. 2b), 4/20 NER genes had an increase in gene expression at the time of relapse. The pattern of expression of the overall pathway was not upregulated, in fact, the pathway was significantly downregulated (*P* = .007). When the datasets were combined the, early relapsers exhibited seven upregulated genes, and the pathway was not significantly upregulated (*P* = .180, Fig. 2c*).*

In the late relapsing subgroup of the Staal dataset, 16/20 NER genes were upregulated at relapse compared with diagnosis (Fig. 2d). Upregulation in the pathway was significant (*P* = .007) and nine genes were individually significantly overexpressed. In the Hogan dataset (Fig. 2e), 16/20 NER genes were overexpressed at relapse compared with diagnosis (*P* = .007), and eight genes were individually significantly overexpressed. In this late relapsing subgroup, this effect occurred in both datasets. Between the two datasets some genes were conserved in expression dysregulation while others were not. For example, *RPA1* was highly overexpressed upon relapse in both datasets, whereas *ERCC6* was not overexpressed in either analysis. However, *GTF2H3* was highly overexpressed in the Hogan cohort, but not overexpressed in the Staal data. The datasets combined (Fig. 2f) showed 16/20 NER genes were overexpressed at recurrence (*P* = .007) with 11 NER genes showed individual significance.

### Time of diagnosis

#### Early vs. late relapsed patients

**Fig. 3:**
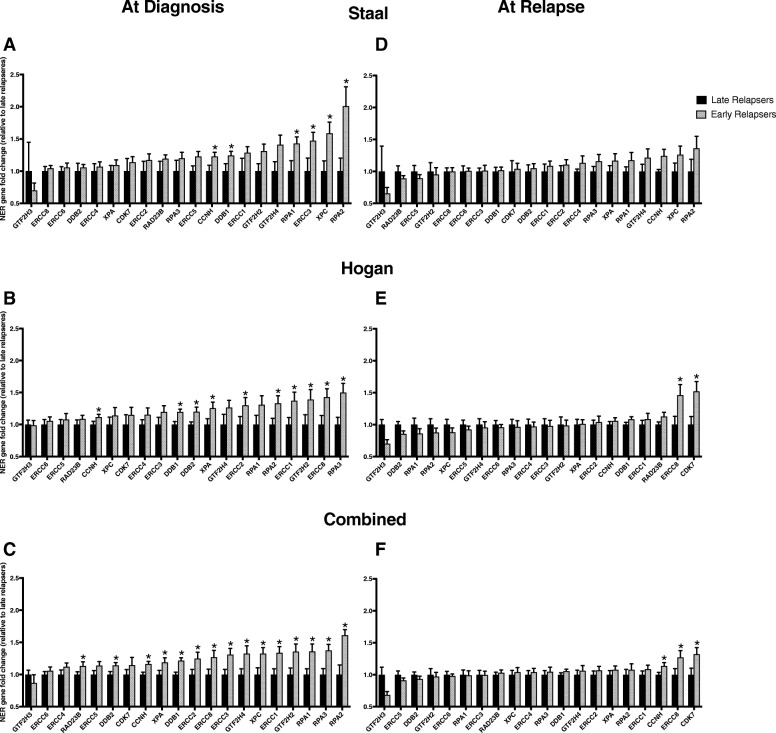
NER gene expression of Early compared to Late Relapsers at diagnosis and at relapse. At diagnosis: (A) In Staal dataset 19 genes were increased in early (gray, *n* = 19) compared to late relapsers (black, *n* = 8) (*P* < .001). 6 genes were individually significantly overexpressed: *CCNH* (*P* = .031), *DDB1* (*P* = .034), *RPA1* (*P* = .015), *ERCC3* (*P* = .031), *XPC* (*P* = .027) and *RPA2* (*P* = 0.024). (B) In the Hogan dataset, 19 genes were overexpressed in early (*n* = 27) compared to late relapsers (*n* = 22, *P* < .001). 10 genes showed significant increases in expression: *CCNH* (*P* = .039), *DDB1* (*P* = .002), *DDB2* (*P* = .014), *XPA* (*P* = .031), *ERCC2* (*P* = .048), *RPA2* (*P* = .019), *ERCC1* (*P* = .023), *GTF2H2* (*P* = .041), *ERCC8* (*P* = .014), and *RPA3* (*P* = .005). (C) In the combined dataset, 19 genes were increased in early (*n* = 46) compared to late relapsers (*n* = 30, *P* < .001). 15 genes were significantly overexpressed: *RAD23B* (*P* = .040), *DDB2* (*P* = .020), *CCNH* (*P* = .003), *XPA* (*P* = .029), *DBB1* (*P* < .001), *ERCC2* (*P* = .029), *ERCC8* (*P* = .021), *ERCC3* (*P* = .008), *GTF2H4* (*P* = .016), *XPC* (*P* = .018), *ERCC1* (*P* = .007), *GTF2H2* (*P* = .012), *RPA1* (*P* = .008), *RPA3* (*P* = .004), and *RPA2* (*P* = .001). At relapse: (D) In the Staal dataset 16 genes were overexpressed in early (*n* = 19) versus late relapsers (*n* = 8, *P* = .007). No gene was individually significant. (E) In Hogan, 8 genes had an increase in expression in early (*n* = 27) versus late relapsers (*n* = 22, *P* = .371). Two genes were significantly overexpressed: *ERRC8* (*P* = .019) and *CDK7* (*P* = .007). (F) In the combined dataset 13 genes exhibited an increase in expression in early (*n* = 46) versus late relapsers (*n* = 30, *P* = .180). 3 genes were significant: *CCNH* (*P* = .031), *ERCC8* (*P* = .038), and *CDK7* (*P* = .018). * indicates *P* < .05 in a one tailed *t*-test

The late relapsers manifested an increase in NER expression from diagnosis to relapse. In contrast, the early relapsers did not change. To investigate the relative levels of expression in these disparate groups at the time of diagnosis, we performed further subset analyses. In the Staal dataset at diagnosis, 19/20 NER genes were upregulated in the early relapse group relative to the late relapse group (Fig. 3a). Therefore, the NER pathway was already significantly overexpressed in the early relapsers compared to late relapsers at diagnosis (*P* < .001) with six genes individually significantly overexpressed. The Hogan dataset showed a similar trend for early relapsers (Fig. 3b), with 19/20 NER genes overexpressed compared to late relapse group; the upregulation of the entire NER pathway was statistically significant (*P* < .001). Ten genes showed individually significant increases in expression. In the combined dataset at the time of diagnosis (Fig. 3c), 19/20 NER genes (all but *GTF2H3*) were upregulated in the early relapsed patients in comparison to late relapsed patients (*P* < .001) and 15 genes were individually significantly overexpressed.

### Time of relapse

#### Early vs. late relapsed patients

An increase in expression of NER genes in late relapsers from diagnosis to relapse (Figs. 2d, e, f) could be due to clonal selection, induction of the NER pathway, or some combination of the two. To determine whether the extent of NER gene expression at relapse was similar in both types of relapsers, we examined NER gene expression at the time of recurrence. In the Staal dataset (Fig. 3d), the NER pathway was significantly upregulated (*P* = .007) with 16/20 genes overexpressed in early relapsers compared with late relapsers at the time of relapse. In the Hogan dataset (Fig. 3e), 8/20 genes had an increase in gene expression in early relapsers compared to late relapsers at the time of relapse, and no significant upregulation in the pathway (*P* = .371). Similarly, in the combined dataset (Fig. 3f), 13/20 genes exhibited an increase in gene expression (*P* = 0.180). At the time of recurrence, the NER gene expression of early and late relapsers was similar whether it had been pre-existing or not. Analysis of NER gene expression in T-ALL was also completed, although only available in a single data set, and can be found in Additional file 4: Figure S3. Our results suggest that NER gene upregulation is associated with relapse in late relapsers but not in early relapsers in which the NER expression at diagnosis is already high.

### Establishment and analysis of NER scores

In order to reduce effects on the pathway to a single value, we created a new metric, the “NER score,” consisting of the average expression of all 20 NER genes normalized to the average expression of all patients at relapse (within Staal or Hogan datasets, since there is no consist significant difference in NER gene expression between early and late relapsers at relapse). NER scores were centered on 1 for relapsing patients, but were often below 1 at diagnosis. In Table 1, the pattern of significant overexpression of NER genes in early relapsers vs. late relapsers at diagnosis can be seen in both sets of data. The upregulation of NER genes in late relapsers from diagnosis to relapse is a trend in the Staal dataset (*P* = .064), but reached significance in the Hogan data set (*P* < .001). The NER score also consistently reflected the lack of change in NER gene expression in early relapsers.

**Table 1:** Analysis of in NER score in ALL in terms of early or late relapsing subgroups

Early Relapsers	NER Scores		
	Diagnosis	Relapse	P^*^
Hogan	1.09 ± 0.06	1.01 ± 0.05	.123
Staal (B + T)	0.99 ± 0.03	1.00 ± 0.04	.749
Staal (B only)	1.01 ± 0.04	1.01 ± 0.05	.974
Hogan + Staal (B + T)	1.04 ± 0.04	1.00 ± 0.03	.266
Hogan + Staal (B only)	1.06 ± 0.04	1.01 ± 0.03	.170
Late Relapsers			
	Diagnosis	Relapse	P^*^
Hogan	0.87 ± 0.06	0.99 ± 0.05	<.001
Staal (B + T)	0.89 ± 0.07	1.01 ± 0.05	.050
Staal (B only)	0.83 ± 0.06	0.96 ± 0.03	.064
Hogan + Staal (B + T)	0.88 ± 0.05	0.99 ± 0.04	<.001
Hogan + Staal (B only)	0.86 ± 0.05	0.98 ± 0.04	<.001
Diagnosis			
	Late Relapsers	Early Relapsers	P^*^
Hogan	0.87 ± 0.06	1.09 ± 0.06	.019
Staal (B + T)	0.89 ± 0.07	0.99 ± 0.03	.197
Staal (B only)	0.83 ± 0.06	1.01 ± 0.04	.031
Hogan + Staal (B + T)	0.88 ± 0.05	1.04 ± 0.04	.009
Hogan + Staal (B only)	0.86 ± 0.05	1.06 ± 0.04	.003
Relapse			
	Late Relapsers	Early Relapsers	P^*^
Hogan	0.99 ± 0.05	1.01 ± 0.05	.800
Staal (B + T)	1.01 ± 0.05	1.00 ± 0.04	.927
Staal (B only)	0.96 ± 0.03	1.01 ± 0.05	.503
Hogan + Staal (B + T)	0.99 ± 0.04	1.00 ± 0.03	.865
Hogan + Staal (B only)	0.98 ± 0.04	1.01 ± 0.03	.599

^*^P value based on students *t*-test in either diagnosis versus relapse or early relapsers versus late relapsers

### Stratification of clinical outcome based on NER gene expression at diagnosis

**Fig. 4:**
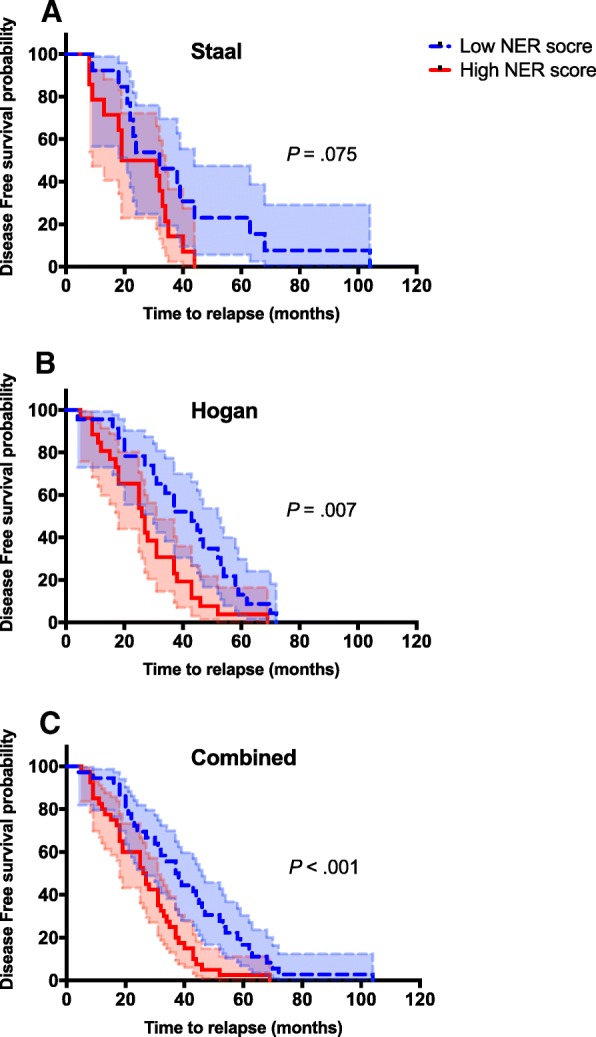
Disease-free survival curves in precursor-B-ALL at the time of diagnosis based on NER score. Three Kaplan Meier plots were generated for Staal (A), Hogan (B), and the combined dataset (C). The shaded area represents the 95% CI for each curve. *P* values using the log-rank test for each dataset comparing the two curves are shown on each plot. (A) In the Staal dataset, the median disease-free survival time was 32 months for the low NER score cohort (*n* = 13) and 25 months in the high NER score cohort (*n* = 14). Log-rank HR for the high NER score cohort relative to the low NER score cohort was 1.869, 95% CI (0.854–4.091). (B) In the Hogan dataset, the median disease-free survival time was 43 months for the low NER score cohort (*n* = 23) and 26.5 months in the high NER score cohort (*n* = 26). Log-rank HR for the high NER score cohort relative to the low NER score cohort was 2.034, 95% CI (1.344–3.648). (C) In the combined dataset, the median disease-free survival time was 37.5 months for the low NER score cohort (*n* = 36) and 26.5 months for the high NER score cohort (*n* = 40). Log-rank HR for the high NER score cohort relative to the low NER score cohort was 2.008, 95% CI (1.256–3.211)

We then stratified the patients into high and low NER expression subgroups at the time of diagnosis to create KM plots using the NER score. In the Staal dataset (*n* = 27) (Fig. 4a) NER gene expression was not correlated with the length of disease-free survival, however it did approach significance (*P* = .075). In both the Hogan (*n* = 49) (Fig. 4b) and combined (*n* = 76) (Fig. 4c) datasets, NER gene expression at the time of diagnosis was significantly correlated with the length of disease-free survival (*P* = .007 and < .001, respectively).

In the Staal dataset, the median disease-free survival time was 32 months for the low NER score cohort (*n* = 13) and 25 months for the high NER score cohort (*n* = 14). The HR for the high NER score cohort relative to the low NER score cohort was 1.869, 95% confidence interval (CI) (0.854–4.091). In the Hogan dataset (n = 49), the median disease-free survival time was 43 months for the low NER score cohort (*n* = 23) and 26.5 months in the high NER score cohort (*n* = 26). HR for the high NER score cohort relative to the low NER score cohort was 2.034, 95% CI (1.344–3.648). In the combined dataset, the median disease-free survival time was 37.5 months for the low NER score cohort (*n* = 36) and 26.5 months for the high NER score cohort (*n* = 40). HR for the high NER score cohort relative to the low NER score cohort was 2.008, 95% CI (1.256–3.211). We also examined the effects of adding the T-ALL subgroup to the combined dataset (Additional file 5: Figure S4). When compared to the cytogenetic factors, we found no correlation within these data sets and NER score (*P* = .555, r^2^ = 0.004). NER score did, however, correlate with time to relapse (*P* < .001, r^2^ = 0.159). KM plots using cytogenetic markers showed that children with poor cytogenetic markers in ALL hadthe shortest time to relapse. When combined with NER score, disease-free survival is shortest and significant in children with high NER score and poor cytogenetic markers compared to those with low NER score and favorable cytogenetics (Additional file 2: Figure S1 and Additional file 3: Figure S2).

**Fig. 5:**
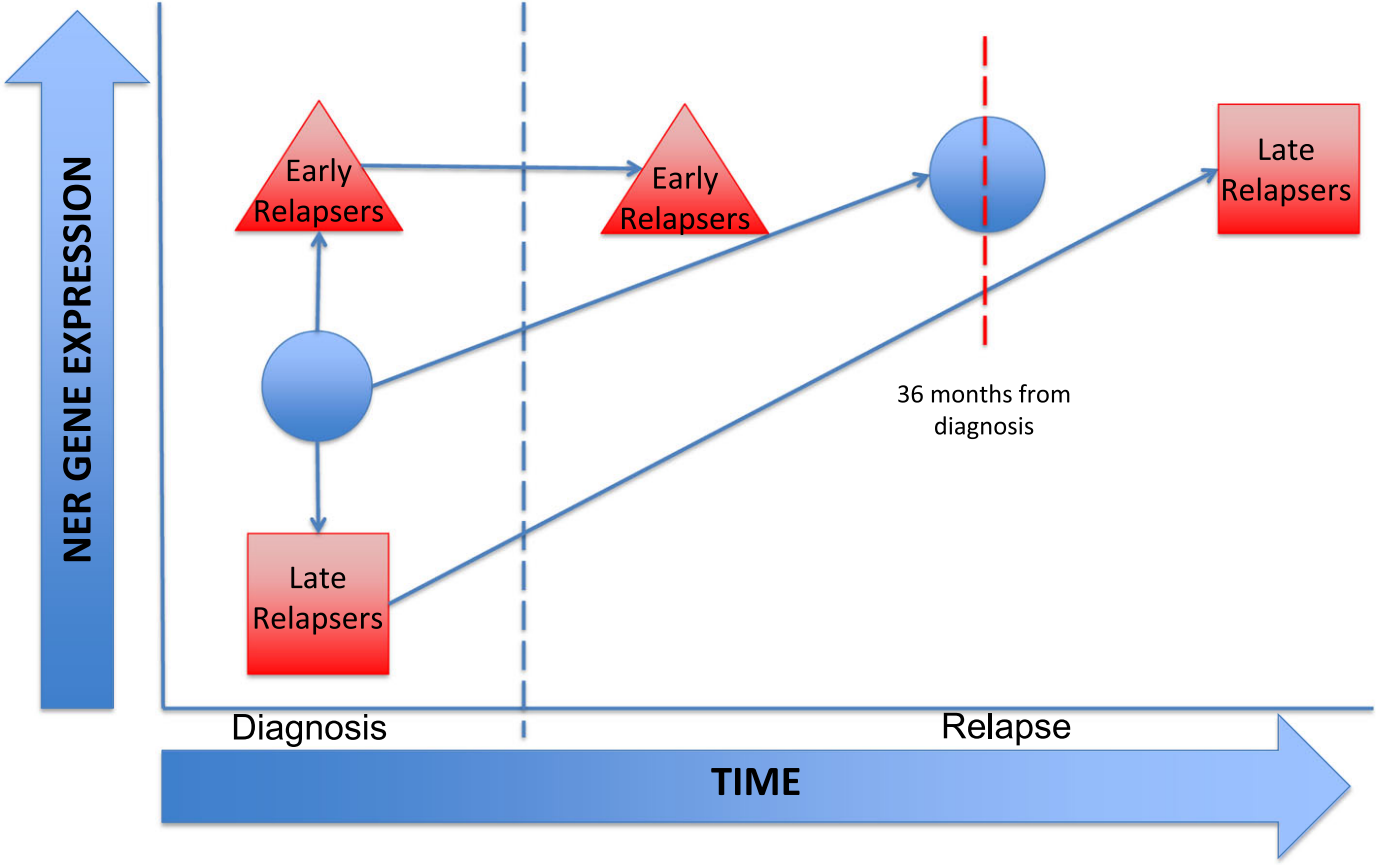
Pediatric precursor-B-ALL relapse model in the context of NER gene expression. Red Triangles represent the early relapse subgroup (Staal *n* = 19, Hogan *n* = 27, total = 46). Red squares represent the late relapse subgroup (Staal *n* = 8, Hogan *n* = 22, total = 30). Blue circles represent all subgroups together (Staal *n* = 27, Hogan *n* = 49, total = 76). Upon subset analysis based on time to relapse, patients that relapsed in under 36 months (“early relapsers”) had significantly higher NER gene expression at time of diagnosis. In contrast, the patients that relapsed in greater than or equal to 36 months (“late relapsers”) had a significantly lower initial NER gene expression at diagnosis. Early relapsers did not change from diagnosis to relapse in overall NER gene expression. The late relapsers have a significant increase in NER gene expression from diagnosis to relapse. At relapse, all patients have the same level of NER gene expression. We can distinguish patients at diagnosis who will relapse early versus those who will relapse late as their gene expression separates them. We propose that NER gene expression can be used as an indicator of early vs. late relapse at the time of diagnosis

NER score data given in Table 1 and the data from this study support the model proposed in Fig. 5. In this model, patients with relatively low initial NER gene expression are more likely to relapse after three years, and their NER pathway gene expression is consistently increased at relapse relative to diagnosis. Patients with relatively high initial NER gene expression relapse in less than three years, without any significant post-diagnostic change in NER gene expression. We tested this model by defining each patient pair as having an increase or decrease in NER score from diagnosis to relapse (Additional file 6: Table S2). Overall, diagnosis-relapse pairs of the early relapsing subgroups in Hogan and Staal, showed no skewing of NER scores. In contrast, in late relapsing subgroups, there was significant skewing with an increasing NER score in the Hogan and combined datasets. This increased skewing also approached significance in the Staal dataset alone. The distributions were significantly different for the early vs. late relapsing subgroups in the Hogan data but were not significant in the Staal data. Patient pair analysis did not change by immunophenotype.

## Discussion

In this study, for the first time, we demonstrate the relationship between NER gene expression and ALL relapse. Our data suggest that the acquisition of increased NER gene expression is an underlying mechanism of both the initial drug resistance (early relapsers) and eventual relapse in ALL (late relapsers). This study was generated from microarray gene expression data for the NER pathway, and enhanced expression of NER genes may not guarantee increased repair capacity or function. However, increased function of NER is highly associated with increased gene expression [32]. Furthermore, changes in NER gene expression that were associated with changes in function were not associated with cell proliferation in breast cancer, indicating that changes in NER are not due to increased cell division [32].

Results from this study may provide a mechanistic basis for a well-established prognostic factor in ALL, time to remission [33]. Cancer cells with low NER may be more susceptible to genotoxic therapy and be eradicated quickly, with fewer cells surviving the initial therapy, leading to relapse after a long remission. Cells with relatively high DNA repair may be more refractory to treatment, with shorter remission times because they are metabolically ready to relapse. This suggests that patients with high initial DNA repair gene expression levels may be better served to consider non-genotoxic types of treatment, such as targeted agents or immunotherapy.

In terms of using these results prognostically, the KM plots suggest that they are relevant and potentially useful. The NER score considers all the canonical genes in the pathway together, instead of just the most significant, which may vary from patient to patient, making it more reliable. Based on this NER score, we find that there are two types of ALL patients, those who display relatively high NER scores and those who display relatively low NER scores at diagnosis. Patients with high NER scores are at greatest risk for early relapse by over 2-fold and 50% of these patients have relapsed 11 months earlier than those with low NER scores. Knowing which patients are at risk for early relapse may improve clinical outcomes, but this study is limited by not knowing the NER gene expression levels in non-relapsing ALL.

The NER score provides a prognostic value that is inclusive of all the canonical genes in the pathway. Useful cytogenetic markers are rare and do not always manifest in ALL patients, as evidenced by these databases where only a small number showed poor cytogenetic markers. When we combined these variables, we found that the NER metric improves prognostic ability, and can be obtained for every patient. However, we were limited by the low n overall, which, once stratified by NER and cytogenetics, leaves only four children in the high NER/poor cytogenetics cohort (Additional file 3: Figure S2).

Much is being done to understand disease progression in childhood leukemia, especially regarding changes in cytogenetics, immunophenotype, and other prognostic variables. Only one of these two datasets included information on such additional variables as white blood cell count, and in that dataset, white blood cell count did not correlate with NER score or timing of relapse.

Since T-ALL patients were present in only one of the datasets (meaning we have no validation set for this group), there was a low number of such patients, and they were highly skewed with regard to the time of relapse. We therefore cannot say whether our results apply to this group. Analysis of the Staal data with and without inclusion of the T-ALL subgroup or the combined data with and without these patients does not reveal a clear and consistent pattern. If there is a relationship between NER gene expression and relapse in T-ALL, it may depend on a unique definition of early and late relapse. These results showed that NER plays a role in the relapse timing of precursor-B-ALL, however, our study of these two databases is limited by the low patient number. Our study must be interpreted in the context of this limitation.

## Conclusion

The NER gene expression of relapsing precursor-B-ALL children showed distinct results depending on the timing of relapse. This was first analyzed in one publicly available database and then confirmed in a second. Children who relapsed early (< 36 months) have significantly higher NER gene expression than those who relapsed later (≥36 months). This is clinically relevant, as precursor-B-ALL children with higher NER scores at diagnosis are over two times more likely to undergo such early relapse, HR 2.008, 95% CI (1.256–3.211). The NER score may provide a mechanism for “time to remission”, a known prognostic factor in ALL, and a rationale for differential treatment.

## Additional files

Additional file 1: **Table S1.** Patient information available from the two databases. (PDF 149 kb)

Additional file 2: **Figure S1.** Linear regression models. (A) Linear regression model of cytogenetic abnormalities versus NER score. Staal and Hogan databases were combined, and cytogenetic abnormalities were categorized based on the prognosis a value of 0 = normal, 1 = favorable prognosis, and 2 = unfavorable prognosis. Linear regression was then done to compare this with the NER score for each precursor-B-ALL child. We found that there was no significant correlation between these two factors (*P* = .555, r^2^ = 0.005). (B) Linear regression model of Cytogenetic abnormalities and NER score versus time to relapse. Staal and Hogan databases were combined, and cytogenetic abnormalities were categorized based on the prognosis a value of 0 = normal, 1 = favorable prognosis, and 2 = unfavorable prognosis. NER score and cytogenetic prognoses were then compared against time to relapse (in months). In these databases cytogenetics was not correlated with time to relapse and the slope of the line was not significant (*P* = .349, r^2^ = 0.119). The NER score was significantly correlated (*P* < .001, r^2^ = 0.159) in the negative direction, therefore, the higher the NER score the earlier the relapse. (PDF 58 kb)

Additional file 3: **Figure S2.** KM plots of cytogenetic abnormalities and NER scores. (A) KM plot of cytogenetic abnormalities. Staal and Hogan databases were combined, and cytogenetic abnormalities were categorized based on the prognosis a value of normal, favorable prognosis, and unfavorable prognosis, then KM plots were created based on time to relapse. The KM plots are significant based on log-rank (Mantel-Cox) test (*P* < .001) and the curves are significantly different from each other (*P* < .001, Gehan-Breslow-Wilcoxon test). However, we are limited to low patient numbers, for example the unfavorable category only contains 5 patients. (B) KM plot of Cytogenetic abnormalities and NER scores combined. Staal and Hogan databases were combined, and cytogenetic abnormalities were categorized based on the prognosis value and NER score: Low NER score and favorable prognostic, low NER score regardless of cytogenetic prognosis, high NER score and poor prognostic, high NER score regardless of cytogenetic prognosis, then KM plots were created based on time to relapse. The KM plots are significant based on log-rank (Mantel-Cox) test (*P* < .001) and the curves are significantly different from each other (*p* < 0.001, Gehan-Breslow-Wilcoxon test). However, we are limited to low patient numbers, for example the high NER score and poor cytogenetic category only contains 4 patients. (PDF 115 kb)

Additional File 4: **Figure S3.** Comparative Analysis of the 20 Canonical NER genes in the Staal dataset of only T-ALL patients. (A) In the early relapsers subgroup (*n* = 12) 12 genes were overexpressed at the time of relapse (gray bars, *P* = .371) versus diagnosis (black bars). One gene was significantly over expressed: *ERCC1* (*P* = .029). (B) In the late relapsers subgroup (n = 1), 15 genes were overexpressed at relapse versus diagnosis (*P* = .025), because this group consists of a single patient statistical analysis at the gene level could not be assessed. (C) At diagnosis the early relapsers (gray bars, *n* = 13) versus late relapsers (black bars, n = 1), 10 genes were upregulated (*P* = 1.000). 2 genes were individually significantly overexpressed: *CDK7* (*P* = .038) and *ERCC6* (*P* = .040). (D) At relapse in early relapsers versus late relapsers, 2 genes were upregulated and 18 were downregulated so the pathway was significantly downregulated (*P* < .001). 2 genes were individually significantly overexpressed: *ERCC6* (*P* = .041) and *GTF2H3* (*P* = .032). (PDF 95 kb)

Additional file 5: **Figure S4.** Disease-free survival curves of overall NER gene expression at diagnosis in combined T-ALL and precursor-B-ALL. For the Low NER score group (dashed blue) versus high NER score group (solid red), 2 Kaplan Meier plots were generated for (A) Staal and (B) combined datasets, regardless of immunotype. The shaded area represents 95% confidence interval (CI) for each curve. *P* values using log-rank test for each data set comparing the two curves are shown on each plot. (A) In the Staal dataset, the median disease-free survival time was 19.5 months for the low NER score cohort (*n* = 22) and 19 months in the high NER score cohort (*n* = 19). Log-rank Hazard Ratio (HR) for the high NER score cohort relative to the low NER score cohort was 1.162, 95% CI (0.626–2.158). (B) In the combined dataset, the median disease-free survival time was 31 months for the low NER score cohort (*n* = 45) and 25 months for the high cohort (n = 45). Log rank HR for the high NER score cohort relative to the low NER score cohort was 1.602, 95% CI (1.048–2.450). (PDF 81 kb)

Additional file 6: **Table S2.** Direction of matched diagnosis to relapse change of NER scores in relapsing ALL children. (PDF 53 kb)

## Abbreviations

ALL: Acute lymphoblastic leukemia
CI: Confidence interval
HR: Hazard ratio
KM: Kaplan-Meier
NER: Nucleotide excision repair
NHEJ: Non-homologous end joining
