# Supplementary material for: Nucleotide excision repair is a predictor of early relapse in pediatric acute lymphoblastic leukemia
Source: BMC Med Genomics. 2018 Oct 30;11:95. doi: 10.1186/s12920-018-0422-2 (PMC6208034; doi:10.1186/s12920-018-0422-2)
Supplement: Supplementary file 1 — Table S1. Patient information available from the two databases. (PDF 149 kb) [file 12920_2018_422_MOESM1_ESM.pdf]

Data set	Relapse timing	Immunotype	Months to relapse	Days to relapse	Age at diagnosis (Days)	Age at relapse (Days)	Cytogenetics	Relpase type	Subtype	WBC (x1e9/L) at diagnosis	WBC (x1e9/L) at relapse
GSE18497	Early	Precursor-B-ALL	22	674	745	1419		C	Pre-B-ALL	71.1	3.1
GSE18497	Late	Precursor-B-ALL	44	1345	830	2175		IBM	Pre-B-ALL	227	5.8
GSE18497	Late	Precursor-B-ALL	39	1214	1010	2224	High hyperdiploid	IBM	Common ALL	41.9	4.1
GSE18497	Early	Precursor-B-ALL	24	755	2739	3494	High hyperdiploid	IBM	Common ALL	50.5	2.3
GSE18497	Early	Precursor-B-ALL	18	546	643	1189	TEL-AML1+	IBM	Pre-B-ALL	74.1	74.3
GSE18497	Early	Precursor-B-ALL	23	721	1006	1727		IBM	Common ALL	3.5	3.5
GSE18497	Early	Precursor-B-ALL	9	295	1079	1374	High hyperdiploid	IBM	Common ALL	no data	0.6
GSE18497	Early	Precursor-B-ALL	8	272	3394	3666	TEL-AML1+	IBM	Pro-B-ALL	386.1	12.3
GSE18497	Late	Precursor-B-ALL	44	1359	1298	2657	t(4;11)(q21;q23) MLL-AFF1	C	Common ALL	9	9.7
GSE18497	Late	Precursor-B-ALL	104	3191	1769	4960		IBM	Common ALL	21.5	2.8
GSE18497	Early	Precursor-B-ALL	35	1062	500	1562		IBM	Pre-B-ALL	10.4	4.9
GSE18497	Early	Precursor-B-ALL	32	963	996	1959		IBM	Common ALL	14.7	17
GSE18497	Early	Precursor-B-ALL	32	995	4627	5622		C	Common ALL	13.3	27
GSE18497	Early	Precursor-B-ALL	31	953	603	1556		IBM	Common ALL	47.4	24.9
GSE18497	Late	Precursor-B-ALL	63	1932	35923	37855		IBM	Pre-B-ALL	191.6	10.8
GSE18497	Early	Precursor-B-ALL	8	244	5008	5252		IBM	Common ALL	195.5	2.9
GSE18497	Late	Precursor-B-ALL	40	1192	941	2133		IBM	Common ALL	64.5	7.2
GSE18497	Early	Precursor-B-ALL	19	579	1750	2329		IBM	Common ALL	17	6.6
GSE18497	Early	Precursor-B-ALL	33	976	2727	3703	High hyperdiploid	IBM	Common ALL	18.8	17.5
GSE18497	Late	Precursor-B-ALL	68	2041	2626	4667		IBM	Pro-B-ALL	23.7	46.8
GSE18497	Early	Precursor-B-ALL	34	1022	3580	4602		IBM	Common ALL	163.4	68.5
GSE18497	Early	Precursor-B-ALL	19	577	4688	5265	High hyperdiploid	IBM	Pro-B-ALL	12.3	1.4
GSE18497	Early	Precursor-B-ALL	18	539	3710	4249	High hyperdiploid	IBM	Common ALL	187	no data
GSE18497	Early	Precursor-B-ALL	21	628	873	1501	t(9;22)	C	Common ALL	101	5.9
GSE18497	Late	Precursor-B-ALL	38	1185	3657	4842	t(9;22)	IBM	Common ALL	27.4	14.9
GSE18497	Early	Precursor-B-ALL	13	379	3783	4162		IBM	Pre-B-ALL	5.9	3.3
GSE18497	Early	Precursor-B-ALL	9	282	1056	1338	Hypodiploid	IBM	Common ALL	149.0	no data
GSE18497	Early	T-ALL	12	373	2600	2973		C		85	8.8
GSE18497	Early	T-ALL	13	412	4018	4430		IBM		149.4	94
GSE18497	Early	T-ALL	10	329	2256	2585		IBM		68.9	1
GSE18497	Early	T-ALL	7	240	745	985		IBM		580.1	20.2
GSE18497	Early	T-ALL	5	158	2034	2192		IBM		95	36
GSE18497	Early	T-ALL	7	239	2345	2584		IBM		98	38.8
GSE18497	Early	T-ALL	7	224	2230	2454		C		167.2	11.7
GSE18497	Late	T-ALL	38	1126	4061	5187		C		53.9	7.8
GSE18497	Early	T-ALL	14	436	1196	1632	t(10;14)(q24;q11)	IBM		10.6	15.5
GSE18497	Early	T-ALL	13	392	2147	2539		IBM		276	18.1
GSE18497	Early	T-ALL	13	423	4056	4479		IBM		305.0	55.0
GSE18497	Early	T-ALL	14	433	4320	4753		C		384.2	3.9
GSE18497	Early	T-ALL	19	579	2957	3536		IBM		263.0	102.0
GSE18497	Early	T-ALL	7	236	6320	6556	t(11;14)(p13;q11)	IBM		181	25.2
GSE28460	Early	Precursor-B-ALL	20	604	2934	3538	Hyperdiploid				
GSE28460	Early	Precursor-B-ALL	30	917	1854	2771	Normal				
GSE28460	Early	Precursor-B-ALL	4	126	3984	4110	Normal				
GSE28460	Early	Precursor-B-ALL	34	1048	2309	3357	Hyperdiploid				
GSE28460	Late	Precursor-B-ALL	37	1138	1183	2321	Hyperdiploid				
GSE28460	Early	Precursor-B-ALL	16	502	1836	2338	Hyperdiploid				
GSE28460	Late	Precursor-B-ALL	70	2117	963	3080	TEL-AML				
GSE28460	Early	Precursor-B-ALL	27	814	6131	6945	TEL-AML				
GSE28460	Early	Precursor-B-ALL	31	941	1282	2223	TEL-AML				
GSE28460	Early	Precursor-B-ALL	17	529	4173	4702	Normal				
GSE28460	Early	Precursor-B-ALL	15	461	5640	6101	Hyperdiploid				
GSE28460	Early	Precursor-B-ALL	18	541	2378	2919	Normal				
GSE28460	Early	Precursor-B-ALL	31	951	2342	3293	Hyperdiploid- trisomy 4,10				
GSE28460	Early	Precursor-B-ALL	11	333	5620	5953	Normal				

GSE28460	Early	Precursor-B-ALL	18	547	2677	3224	Normal
GSE28460	Early	Precursor-B-ALL	25	776	699	1475	TEL-AML
GSE28460	Early	Precursor-B-ALL	12	372	6685	7057	Normal
GSE28460	Early	Precursor-B-ALL	18	547	454	1001	MLL
GSE28460	Early	Precursor-B-ALL	28	865	4616	5481	Normal
GSE28460	Early	Precursor-B-ALL	9	290	6140	6430	Normal
GSE28460	Early	Precursor-B-ALL	31	935	4756	5691	Normal
GSE28460	Early	Precursor-B-ALL	25	771	853	1624	Normal
GSE28460	Early	Precursor-B-ALL	27	820	1449	2269	TEL-AML
GSE28460	Early	Precursor-B-ALL	27	821	2543	3364	Normal
GSE28460	Early	Precursor-B-ALL	26	788	3872	4660	Normal
GSE28460	Early	Precursor-B-ALL	25	766	6740	7506	Normal
GSE28460	Early	Precursor-B-ALL	5	171	5491	5662	Normal
GSE28460	Early	Precursor-B-ALL	9	293	5906	6199	Normal
GSE28460	Late	Precursor-B-ALL	54	1641	2298	3940	Normal
GSE28460	Late	Precursor-B-ALL	58	1769	1342	3111	Normal
GSE28460	Late	Precursor-B-ALL	72	2179	1971	4150	TEL-AML
GSE28460	Late	Precursor-B-ALL	59	1780	4324	6104	Normal
GSE28460	Late	Precursor-B-ALL	43	1316	2143	3459	Hyperdiploid
GSE28460	Late	Precursor-B-ALL	52	1579	1010	2589	Hyperdiploid
GSE28460	Late	Precursor-B-ALL	47	1428	764	2192	Hyperdiploid
GSE28460	Early	Precursor-B-ALL	20	617	1389	2006	TEL-AML
GSE28460	Late	Precursor-B-ALL	53	1308	1275	2583	TEL-AML
GSE28460	Late	Precursor-B-ALL	62	1865	4863	6728	Normal
GSE28460	Late	Precursor-B-ALL	45	1373	2891	4264	Hyperdiploid
GSE28460	Late	Precursor-B-ALL	46	1389	5853	7242	Normal
GSE28460	Late	Precursor-B-ALL	69	2075	1433	3508	TEL-AML
GSE28460	Late	Precursor-B-ALL	37	1121	1292	2413	TEL-AML
GSE28460	Late	Precursor-B-ALL	52	1575	5759	7334	Normal
GSE28460	Late	Precursor-B-ALL	43	1311	1079	2390	TEL-AML
GSE28460	Late	Precursor-B-ALL	46	1405	6114	7519	Normal
GSE28460	Late	Precursor-B-ALL	37	1138	5213	6351	Normal
GSE28460	Late	Precursor-B-ALL	43	1310	4836	6146	Hyperdiploid- trisomy 4,10,17
GSE28460	Late	Precursor-B-ALL	38	1148	6378	7526	Normal
GSE28460	Late	Precursor-B-ALL	37	1128	3106	4234	Normal

Additional Table 1. Summary of patient data from two publicaly available databases.

Abbreviations: ALL, acute lymphoblastic leukemia; C, combined; IBM, isolated bone marrow
