# Supplementary material for: Nucleotide excision repair is a predictor of early relapse in pediatric acute lymphoblastic leukemia
Source: BMC Med Genomics. 2018 Oct 30;11:95. doi: 10.1186/s12920-018-0422-2 (PMC6208034; doi:10.1186/s12920-018-0422-2)
Supplement: Supplementary file 2 — Figure S1. Linear regression models. (A) Linear regression model of cytogenetic abnormalities versus NER score. Staal and Hogan databases were combined, and cytogenetic abnormalities were categorized based on the prognosis a value of 0 = normal, 1 = favorable prognosis, and 2 = unfavorable prognosis. Linear regression was then done to compare this with the NER score for each precursor-B-ALL child. We found that there was no significant correlation between these two factors (P = .555, r2 = 0.005). (B) Linear regression model of Cytogenetic abnormalities and NER score versus time to relapse. Staal and Hogan databases were combined, and cytogenetic abnormalities were categorized based on the prognosis a value of 0 = normal, 1 = favorable prognosis, and 2 = unfavorable prognosis. NER score and cytogenetic prognoses were then compared against time to relapse (in months). In these databases cytogenetics was not correlated with time to relapse and the slope of the line was not significant (P = .349, r2 = 0.119). The NER score was significantly correlated (P < .001, r2 = 0.159) in the negative direction, therefore, the higher the NER score the earlier the relapse. (PDF 58 kb) [file 12920_2018_422_MOESM2_ESM.pdf]

A

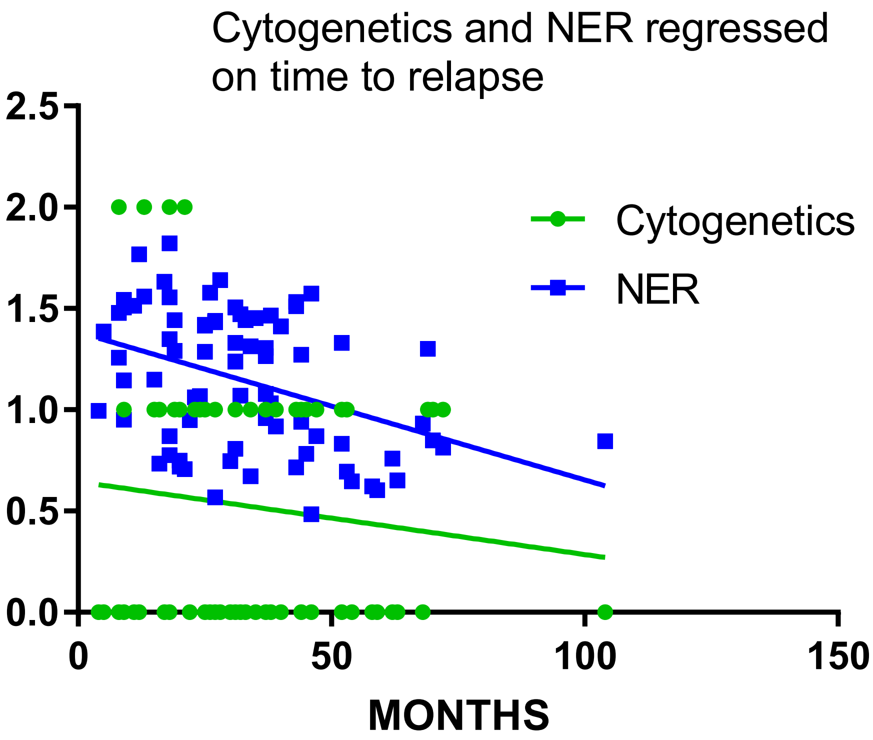

	Cytogenetics	NER
Best-fit values ± SE		
Slope	-0.003595 ± 0.003815	-0.007285 ± 0.001949
Y-intercept	0.6449 ± 0.1447	1.383 ± 0.07391
X-intercept	179.4	189.8
1/slope	-278.2	-137.3
95% Confidence Intervals		
Slope	-0.0112 to 0.004007	-0.01117 to -0.0034
Y-intercept	0.3567 to 0.9331	1.235 to 1.53
X-intercept	77.43 to +infinity	134.7 to 369.5
Goodness of Fit		
R square	0.01186	0.1587
Sy.x	0.6217	0.3177
Is slope significantly non-zero?		
F	0.888	13.96
DFn, DFd	1, 74	1, 74
P value	0.3491	0.0004
Deviation from zero?	Not Significant	Significant
Equation	Y = -0.003595*X + 0.6449	Y = -0.007285*X + 1.383
Data		
Number of X values	76	76
Maximum number of Y replicates	1	1
Total number of values	76	76
Number of missing values	0	0

B

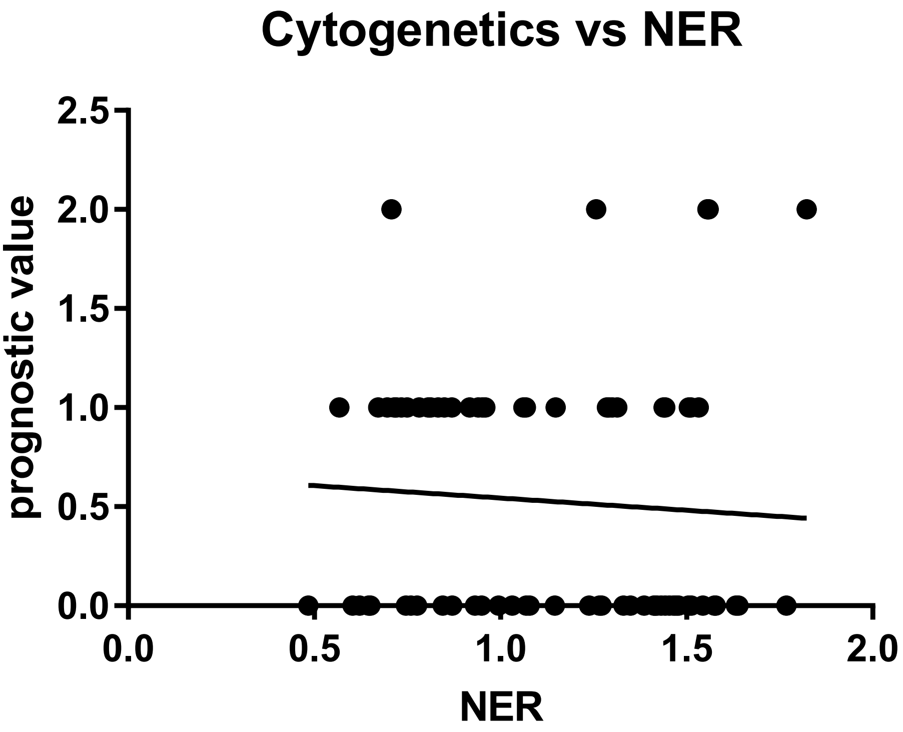

	Cytogenetics
Best-fit values ± SE	
Slope	-0.1242 ± 0.2094
Y-intercept	0.6682 ± 0.2497
X-intercept	5.381
1/slope	-8.053
95% Confidence Intervals	
Slope	-0.5415 to 0.2931
Y-intercept	0.1706 to 1.166
X-intercept	2.067 to +infinity
Goodness of Fit	
R square	0.004729
Sy.x	0.624
Is slope significantly non-zero?	
F	0.3516
DFn, DFd	1, 74
P value	0.5550
Deviation from zero?	Not Significant
Equation	Y = -0.1242*X + 0.6682
Data	
Number of X values	76
Maximum number of Y replicates	1
Total number of values	76
Number of missing values	0
