# Supplementary material for: Nucleotide excision repair is a predictor of early relapse in pediatric acute lymphoblastic leukemia
Source: BMC Med Genomics. 2018 Oct 30;11:95. doi: 10.1186/s12920-018-0422-2 (PMC6208034; doi:10.1186/s12920-018-0422-2)
Supplement: Supplementary file 3 — Figure S2. KM plots of cytogenetic abnormalities and NER scores. (A) KM plot of cytogenetic abnormalities. Staal and Hogan databases were combined, and cytogenetic abnormalities were categorized based on the prognosis a value of normal, favorable prognosis, and unfavorable prognosis, then KM plots were created based on time to relapse. The KM plots are significant based on log-rank (Mantel-Cox) test (P < .001) and the curves are significantly different from each other (P < .001, Gehan-Breslow-Wilcoxon test). However, we are limited to low patient numbers, for example the unfavorable category only contains 5 patients. (B) KM plot of Cytogenetic abnormalities and NER scores combined. Staal and Hogan databases were combined, and cytogenetic abnormalities were categorized based on the prognosis value and NER score: Low NER score and favorable prognostic, low NER score regardless of cytogenetic prognosis, high NER score and poor prognostic, high NER score regardless of cytogenetic prognosis, then KM plots were created based on time to relapse. The KM plots are significant based on log-rank (Mantel-Cox) test (P < .001) and the curves are significantly different from each other (p < 0.001, Gehan-Breslow-Wilcoxon test). However, we are limited to low patient numbers, for example the high NER score and poor cytogenetic category only contains 4 patients. (PDF 115 kb) [file 12920_2018_422_MOESM3_ESM.pdf]

A

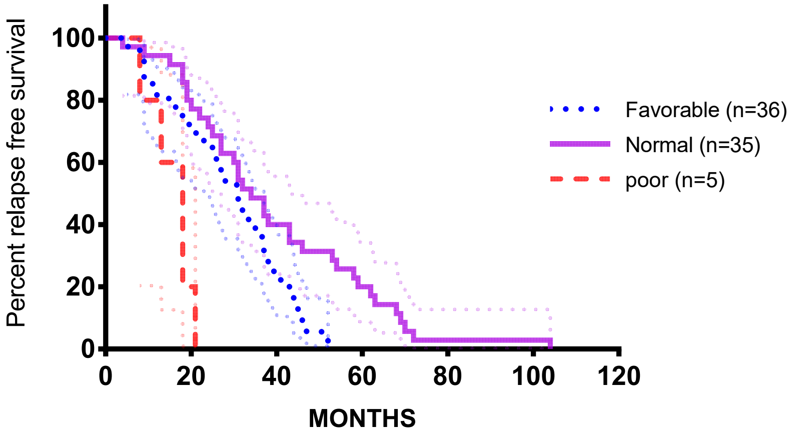

Comparison of Survival Curves	
Log-rank (Mantel-Cox) test (recommended)	
Chi square	20.83
df	2
P value	<0.0001
P value summary	****
Are the survival curves sig different?	Yes
Logrank test for trend (recommended)	
Chi square	1.042
df	1
P value	0.3074
P value summary	ns
Sig. trend?	No
Gehan-Breslow-Wilcoxon test	
Chi square	14.7
df	2
P value	0.0006
P value summary	***
Are the survival curves sig different?	Yes

B

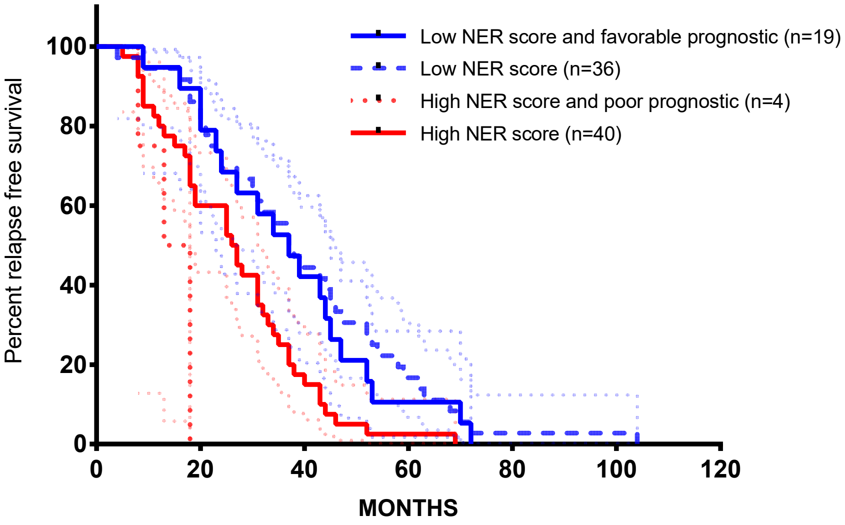

Comparison of Survival Curves	
Log-rank (Mantel-Cox) test (recommended)	
Chi square	27.3
df	3
P value	<0.0001
P value summary	****
Are the survival curves sig different?	Yes
Logrank test for trend (recommended)	
Chi square	10.9
df	1
P value	0.0010
P value summary	***
Sig. trend?	Yes
Gehan-Breslow-Wilcoxon test	
Chi square	23.4
df	3
P value	<0.0001
P value summary	****
Are the survival curves sig different?	Yes
