# Supplementary figures and images for: Nucleotide excision repair is a predictor of early relapse in pediatric acute lymphoblastic leukemia
Source: BMC Med Genomics. 2018 Oct 30;11:95. doi: 10.1186/s12920-018-0422-2 (PMC6208034; doi:10.1186/s12920-018-0422-2)

## A Early relapsers

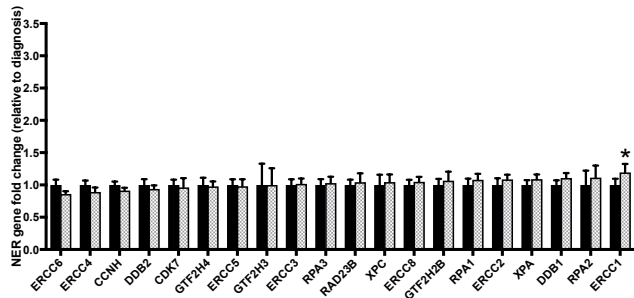

## B Late relapsers

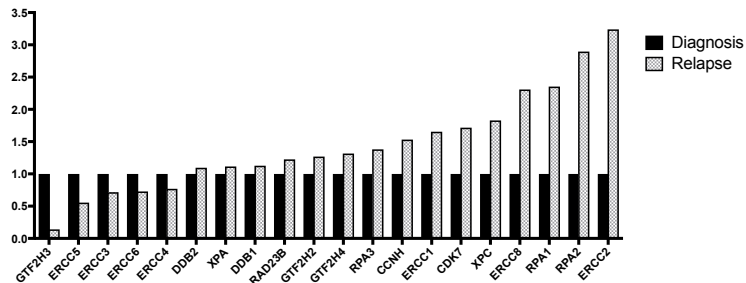

## C At diagnosis

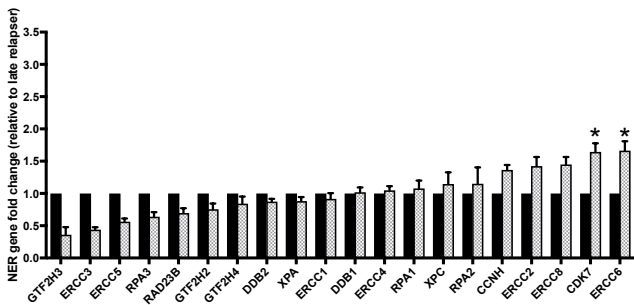

## D At relapse

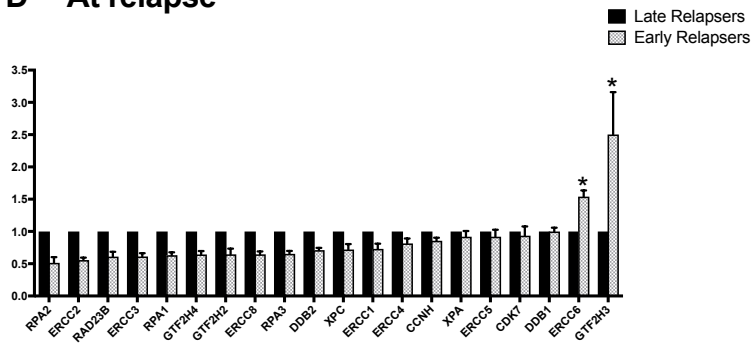

Supplement: Supplementary file 4 — Figure S3. Comparative Analysis of the 20 Canonical NER genes in the Staal dataset of only T-ALL patients. (A) In the early relapsers subgroup (n = 12) 12 genes were overexpressed at the time of relapse (gray bars, P = .371) versus diagnosis (black bars). One gene was significantly over expressed: ERCC1 (P = .029). (B) In the late relapsers subgroup (n = 1), 15 genes were overexpressed at relapse versus diagnosis (P = .025), because this group consists of a single patient statistical analysis at the gene level could not be assessed. (C) At diagnosis the early relapsers (gray bars, n = 13) versus late relapsers (black bars, n = 1), 10 genes were upregulated (P = 1.000). 2 genes were individually significantly overexpressed: CDK7 (P = .038) and ERCC6 (P = .040). (D) At relapse in early relapsers versus late relapsers, 2 genes were upregulated and 18 were downregulated so the pathway was significantly downregulated (P < .001). 2 genes were individually significantly overexpressed: ERCC6 (P = .041) and GTF2H3 (P = .032). (PDF 95 kb) [file 12920_2018_422_MOESM4_ESM.pdf]

**A**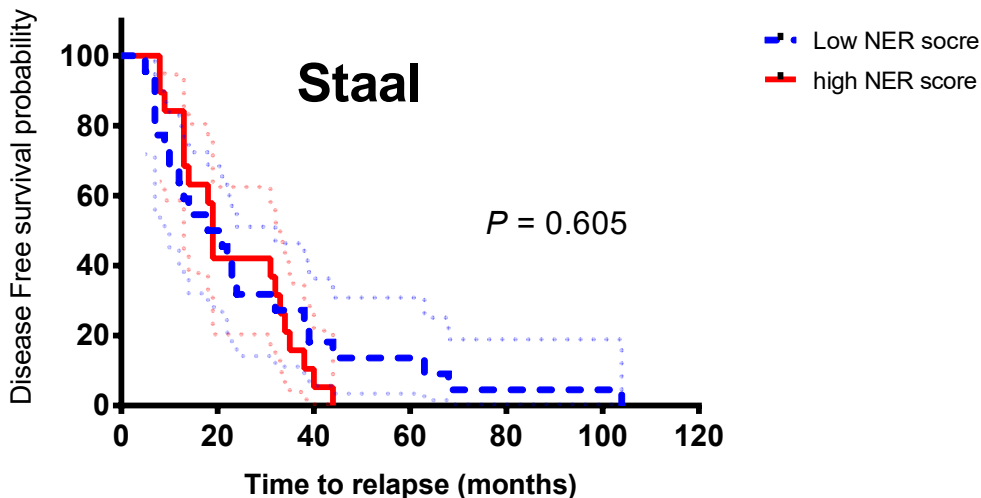**B**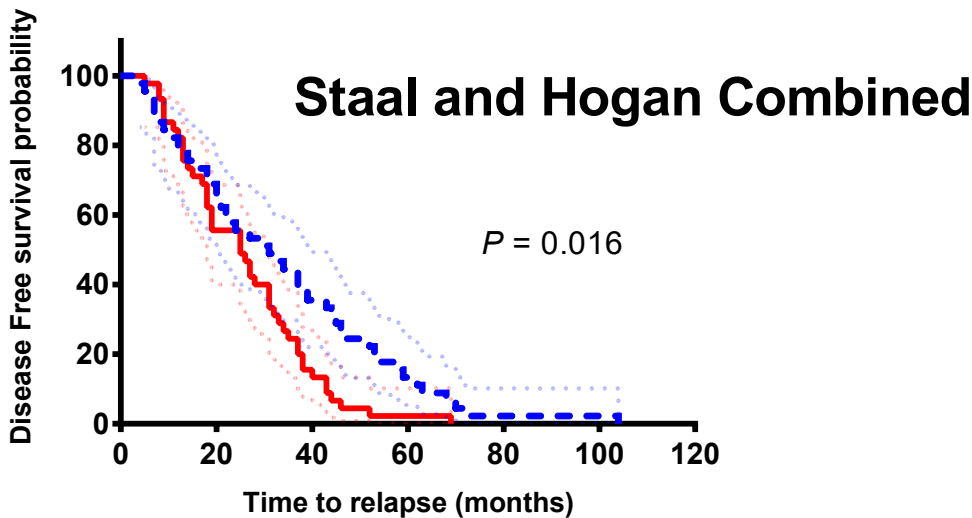

Supplement: Supplementary file 5 — Figure S4. Disease-free survival curves of overall NER gene expression at diagnosis in combined T-ALL and precursor-B-ALL. For the Low NER score group (dashed blue) versus high NER score group (solid red), 2 Kaplan Meier plots were generated for (A) Staal and (B) combined datasets, regardless of immunotype. The shaded area represents 95% confidence interval (CI) for each curve. P values using log-rank test for each data set comparing the two curves are shown on each plot. (A) In the Staal dataset, the median disease-free survival time was 19.5 months for the low NER score cohort (n = 22) and 19 months in the high NER score cohort (n = 19). Log-rank Hazard Ratio (HR) for the high NER score cohort relative to the low NER score cohort was 1.162, 95% CI (0.626–2.158). (B) In the combined dataset, the median disease-free survival time was 31 months for the low NER score cohort (n = 45) and 25 months for the high cohort (n = 45). Log rank HR for the high NER score cohort relative to the low NER score cohort was 1.602, 95% CI (1.048–2.450). (PDF 81 kb) [file 12920_2018_422_MOESM5_ESM.pdf]
