# Supplementary material for: Nucleotide excision repair is a predictor of early relapse in pediatric acute lymphoblastic leukemia
Source: BMC Med Genomics. 2018 Oct 30;11:95. doi: 10.1186/s12920-018-0422-2 (PMC6208034; doi:10.1186/s12920-018-0422-2)
Supplement: Supplementary file 6 — Table S2. Direction of matched diagnosis to relapse change of NER scores in relapsing ALL children. (PDF 53 kb) [file 12920_2018_422_MOESM6_ESM.pdf]

**Table 2**
Direction of matched diagnosis to relapse change of NER score in relapsing ALL

| **Early Relapsers** | **Patient Pairs Change in NER Score Diagnosis to Relapse** | | | |
|---|---|---|---|---|
| | Increase | Decrease | *P** | |
| **Hogan** | 11 | 16 | .336 | |
| **Staal (B+T)** | 17 | 15 | .724 | |
| **Staal (B only)** | 10 | 9 | .819 | |
| **Hogan + Staal (B+T)** | 30 | 29 | .896 | |
| **Hogan + Staal (B only)** | 21 | 25 | .555 | |
| | | | | |
| **Late Relapsers** | **Patient Pairs Change in NER Score Diagnosis to Relapse** | | | |
| | Increase | Decrease | *P** | *P*ᵻ |
| **Hogan** | 17 | 5 | .011 | .019 |
| **Staal (B+T)** | 7 | 2 | .096 | .262 |
| **Staal (B only)** | 6 | 2 | .157 | .405 |
| **Hogan + Staal (B+T)** | 24 | 7 | .003 | .023 |
| **Hogan + Staal (B only)** | 23 | 7 | .004 | .009 |

*Chi-square test comparing observed frequency to theoretical expected frequency for each set
ᵻFishers exact test comparing early vs late relapsers for each set
